# A challenging diagnostic journey of antibody-mediated rejection after lung transplantation in a child: a case report

**DOI:** 10.3389/fmed.2026.1884832

**Published:** 2026-08-17

**Authors:** Francesca Lunardi, Marta Vadori, Monica Loy, Davide Biondini, Giuseppe Maggioni, Ezgi Demirkilic, Andrea Dell’amore, Emanuele Cozzi, Fiorella Calabrese

**Affiliations:** 1 Department of Molecular Medicine, University of Pavia, Pavia, Italy; 2 Fondazione IRCCS Policlinico San Matteo, Pavia, Italy; 3 Department of Cardiac, Thoracic, Vascular Sciences and Public Health, University of Padova, Padova, Italy; 4 Transplant Immunology Laboratory, Institute of Pediatric Research “Città Della Speranza”, Padova, Italy; 5 Padova University Hospital, Padova, Italy

**Keywords:** AMR, case report, histology, lung transplantation (LTx), non-HLA antibodies

## Abstract

Antibody-mediated rejection (AMR) is a serious post–lung transplant (LTx) complication with a challenging diagnosis. Histopathological findings associated with AMR include variable degrees of capillary inflammation, endotheliitis, and diffuse alveolar damage/organizing pneumonia. AMR has been historically related to anti-HLA donor-specific antibodies (DSA), while the role of non-HLA antibodies remains debated. We report the case of a 15-year-old male undergoing bilateral LTx for cystic fibrosis. Twenty days later, the patient developed moderate acute cellular rejection (ACR); in samples not affected by ACR, diffuse edema, capillary inflammation, and high p-S6RP expression scores were observed in macrophages, endothelial and epithelial cells. Anti-HLA class II DSA [maximum median fluorescence intensity (MFI): 10434] supported a diagnosis of mixed cellular/humoral rejection. After steroid therapy, DSA markedly decreased (maximum MFI: 1534), and ACR resolved, whereas persistent capillary inflammation and high p-S6RP expression in all cell types characterized sequential biopsies, raising suspicion for ongoing subclinical AMR. The persistence of these morphological features, together with sustained p-S6RP overexpression, prompted extended immunological testing, which revealed markedly elevated antibodies against endothelin type A receptor and angiotensin II type 1 receptor (>40 U/mL) in the patient follow-up. This case underscores the pivotal role of histopathological findings in multidisciplinary decision-making and highlights the importance of extended immunological screening. Our findings further support the emerging pathogenic relevance of non-HLA antibodies in the development and persistence of AMR in LTx recipients. The patient ultimately died 25 months after transplantation from progressive chronic lung allograft dysfunction.

## Introduction

Antibody-mediated rejection (AMR) represents a major barrier to long-term success in lung transplantation (LTx) and is strongly associated with chronic lung allograft dysfunction (CLAD) and reduced graft survival. Current consensus statements emphasize that AMR diagnosis requires a multidisciplinary approach integrating clinical, immunological, radiological, and histopathological data, with pathologists playing a central role (1, 2). Several morphological abnormalities, including diffuse alveolar damage (DAD), organizing pneumonia (OP), capillary inflammation, endotheliitis, and alveolar septal widening have been associated with AMR (3, 4), although none is pathognomonic, as similar patterns may occur in infections, drug toxicity, or other alloimmune injuries. In solid organ transplantation, HLA donor-specific antibodies (DSA) are recognized as key mediators of AMR. However, the clinical phenotype is variable: some patients with high DSA titers maintain stable graft function, while others develop AMR-like injury without detectable circulating DSA. Proposed explanations include intragraft DSA entrapment and the involvement of non-HLA antibodies, which may target alloantigens or autoantigens such as collagen V, collagen I, K-α1 tubulin, endothelin type A receptor (ETAR), and angiotensin II type 1 receptor (AT1R), acting synergistically with or independently of HLA-DSA (5). Although their clinical significance remains incompletely defined, some studies associate non-HLA antibodies with worse outcomes in lung recipients (6–8). To address this complexity, the STAR 2019 Working Group recommended standardized DSA assessment, investigation of non-HLA antibodies, and integrated diagnostic strategies for improved risk stratification (9). In parallel, immunohistochemical and molecular markers have emerged as diagnostic adjuncts. Among emerging tissue biomarkers, activation of the mammalian target of rapamycin (mTOR) signaling pathway has been implicated in endothelial cell activation and microvascular injury induced by antibody-mediated immune responses. Phosphorylated S6 ribosomal protein (p-S6RP), a downstream effector of mTOR signaling, has therefore emerged as a promising marker of active humoral injury in kidney, heart, and LTx (10–15). This report presents a challenging pediatric lung AMR case marked by discordant clinical, immunological and histopathological findings. Persistent microvascular inflammation and strong, diffuse p-S6RP expression prompted expanded immunological evaluation, ultimately revealing high levels of anti-ETAR and anti-AT1R antibodies in the absence of HLA-DSA.

## Diagnostic methods

According to the 2016 International Society for Heart and Lung Transplantation (ISHLT) consensus on AMR in LTx (2), AMR is classified as clinical or subclinical according to the presence or absence of measurable allograft dysfunction. Subclinical AMR refers to cases without overt graft dysfunction but with immunologic and/or histologic evidence suggestive of antibody-mediated injury. Diagnostic certainty is based on the combination of three components: (1) circulating DSA, (2) histologic features compatible with AMR, and (3) C4d deposition. Definite subclinical AMR requires the presence of all three components, whereas probable subclinical AMR is defined by the presence of two components, including the combination of compatible histology and DSA even in the absence of C4d deposition. Possible subclinical AMR refers to cases in which only one component is present. Diagnostic confidence therefore increases with the accumulation of concordant immunologic, histologic, and complement-related findings.

Immunohistochemistry for p-S6RP was performed on all transbronchial biopsies (TBB) using a rabbit monoclonal anti-p-S6RP antibody (Ser235/236, Cell Signaling Technology), as previously described (12). P-S6RP expression was independently evaluated by experienced lung pathologists (F.L. and F.C.) in endothelial cells, pneumocytes, and macrophages using a semiquantitative score (0–3) based on the extent of strong cytoplasmic staining (0 = absent; 1 = focal; 2 = multifocal; 3 = diffuse). A composite score was obtained by summing the scores of the three cellular compartments, as previously reported (12), with a total score ≥ 7 considered indicative of significant p-S6RP overexpression. Staining results were interpreted in conjunction with the histopathological and clinical findings and according to our previously validated scoring approach.

Anti-HLA Class I and Class II IgG antibodies were measured using a bead-based solid-phase immunoassay on the Luminex platform with commercially available Immucor Lifecodes^®^ kits (Werfen, CT, United States), according to the manufacturer’s instructions. For antibody screening, Lifecodes^®^ LifeScreen Deluxe kits were used. For determination of antibody specificity, Lifecodes^®^ Single Antigen (LSA) Class I and Class II kits were applied. Data acquisition was performed on a Luminex analyzer. In our center, HLA class I and II antibodies are routinely measured on the day of transplantation, 15 days, 1, 3, 6, 9, 12, 18, 24, and 36 months post transplantation. Subsequently, these antibodies are tested annually or in cases with acute or chronic deterioration of graft function, or suspected AMR. All immunological testing is performed at the HLA Laboratory of the Padua University Hospital (Padova, Italy).

Anti-AT1R and anti-ETAR antibodies were not included in the routine monitoring protocol. These antibodies were measured retrospectively by ELISA (CellTrend, Luckenwalde, Germany) on stored pre- and post-transplant serum samples after persistent histological findings prompted an extended immunological investigation. Serum samples corresponded to the same post-transplant time points used for routine HLA antibody monitoring. Each specimen was analyzed in duplicate. According to the manufacturer’s instructions, antibody levels between 10 and 16 U/mL were considered intermediate, whereas values > 17 U/mL were considered positive.

## Case description

### Clinical findings

A 15-year-old boy with cystic fibrosis, diagnosed through neonatal screening, was evaluated for LTx and listed in September 2023.

Until 1 year before transplantation, he maintained acceptable quality of life despite poor growth (BMI 13.3, height: 153 cm, weight: 34 kg). He presented with pancreatic exocrine insufficiency, and glucose intolerance. Over the preceding year, he experienced multiple episodes of infective exacerbations and a progressive decline in lung function, documented by spirometry. He was chronically colonized with *Staphylococcus aureus*, *Stenotrophomonas maltophilia*, *Pseudomonas aeruginosa*, and *Aspergillus flavus*.

Bilateral LTx was performed in October 2023. During his intensive care unit (ICU) stay, frequent airway toileting was required due to thick secretions. Microbiological analyses of sputum and bronchoalveolar lavage (BAL) samples revealed colonization with multidrug-resistant *Pseudomonas aeruginosa*, which was managed with targeted antibiotic therapy. Notably, as the patient had received Varicella Zoster virus (VZV) vaccination the day before transplantation, antiviral prophylaxis with valacyclovir was initiated immediately postoperatively, and VZV-DNA replication was monitored over time.

After hospital discharge, the patient underwent the standard clinical, pathological, and immunological follow-up protocol according to the center’s practice. During follow-up, the patient developed recurrent stenosis of the right bronchial anastomosis, requiring repeated bronchoscopic dilations. Spirometry temporarily improved after each dilation but subsequently deteriorated due to restenosis.

In July 2025, he was admitted to the ICU for respiratory failure and fever. Despite intensive supportive care including high-dose steroids, plasmapheresis, and intravenous immunoglobulins (IVIG), respiratory function progressively worsened. The patient and his family declined re-listing for re-transplantation, and he died on postoperative day 654.

### Therapeutic interventions

**FIGURE 1:**
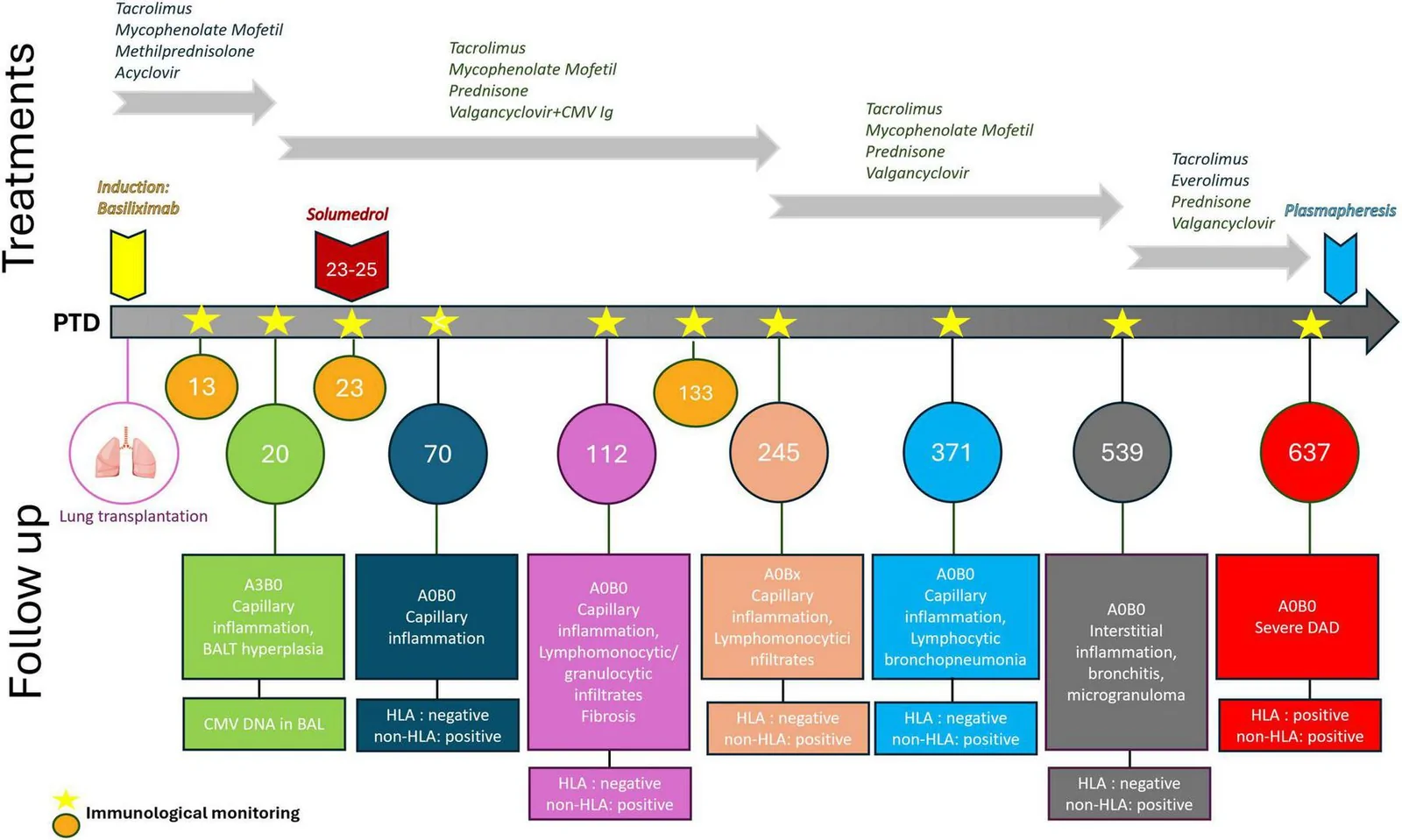
Timeline of stclinical course, treatments, and immunological monitoring after lung transplantation. The timeline summarizes the post-transplant clinical course from PTD 0 to PTD 637. Details of the serial bronchoscopies with histological evaluation are reported. PTD, post-transplant day; BALT, Bronchus-Associated Lymphoid Tissue; DAD, diffuse alveolar damage; BAL, bronchoalveolar lavage.

At induction, the patient received only one dose of basiliximab (20 mg) due to recent vaccination with live-attenuated VZV vaccine. After transplantation, he received a standard triple immunosuppressive regimen with intravenous methylprednisolone (15 mg daily) and intravenous tacrolimus (0.25 mg/day), in combination with oral mycophenolate mofetil (500 mg twice daily). Antiviral prophylaxis was administered with acyclovir (330 mg three times daily). Following the diagnosis of mixed acute cellular rejection (ACR)/AMR at the first follow-up bronchoscopy [post-transplant day (PTD) 20], due to the identification of cytomegalovirus (CMV) DNA in BAL, the patient received high-dose solumedrol only (PTD 23–25) and was discharged with higher immunosuppressive regimen, composed by tacrolimus (5 mg twice daily), mycophenolate mofetil (1 g twice daily), and prednisone (40 mg daily during the first postoperative week, subsequently tapered to 20 mg daily). In addition, combined CMV treatment was initiated with valganciclovir (900 mg twice daily for 15 days, followed by 900 mg once daily) and intravenous CMV-specific immunoglobulin (Cytomegatect^®^, 1,750 IU administered once monthly). At PTD 70, ACR was resolved, and the patient was dismissed with tacrolimus (2 mg twice daily), mycophenolate mofetil (1 g twice daily), prednisone (20 mg daily), valganciclovir (900 mg once daily) and Cytomegatect^®^ (1,750 IU once monthly). At PTD 112, immunosuppression was increased with tacrolimus (4.5 mg twice daily), mycophenolate mofetil (2.5 g daily), prednisone (20 mg daily), valganciclovir (900 mg daily) and Cytomegatect^®^ (1,750 IU once monthly). At PTD 245, prednisone was decreased to 15 mg daily, and tacrolimus was increased to 5 mg daily, then to 6 mg daily at PTD 371. At that time, Cytomegatect^®^ was discontinued, and valganciclovir was initiated at 450 mg three times per week. At PTD 539, mycophenolate mofetil was switched to everolimus 1.5 mg daily; tacrolimus was increased to 9 mg daily, and prednisone was decreased to 7.5 mg daily. The patient remained adherent to the therapeutic regimen throughout the entire follow-up period. Figure 1 reports the timeline of the therapeutic interventions.

### Pathological findings and follow up

**FIGURE 2:**
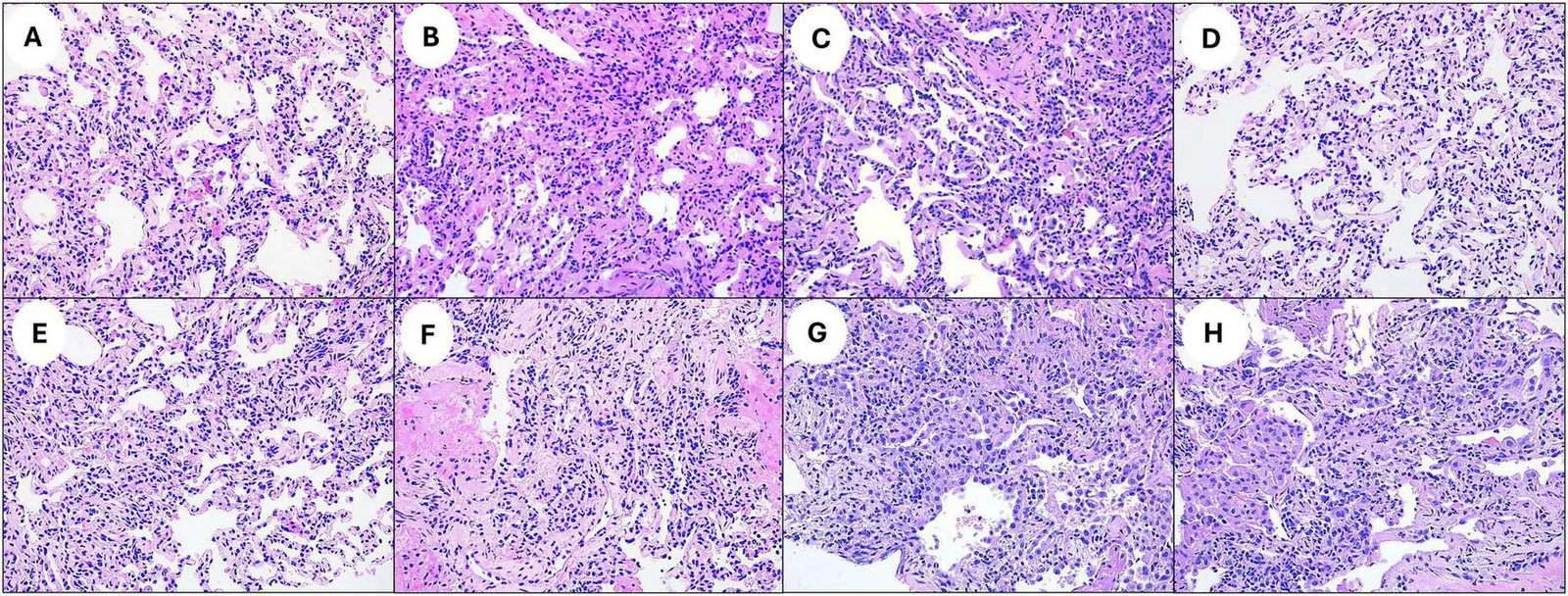
Explanatory images of histological features characterizing TBBs obtained during the whole follow-up of the patient. **(A)** PTD 20, **(B)** PTD 70, **(C)** PTD 112, **(D)** PTD 245, **(E)** PTD 371, **(F)** PTD 539, **(G,H)** PTD 637. In all TBBs lymphomonocytic infiltrate and capillary inflammation are visible. PTD, post-transplant day.

**FIGURE 3:**
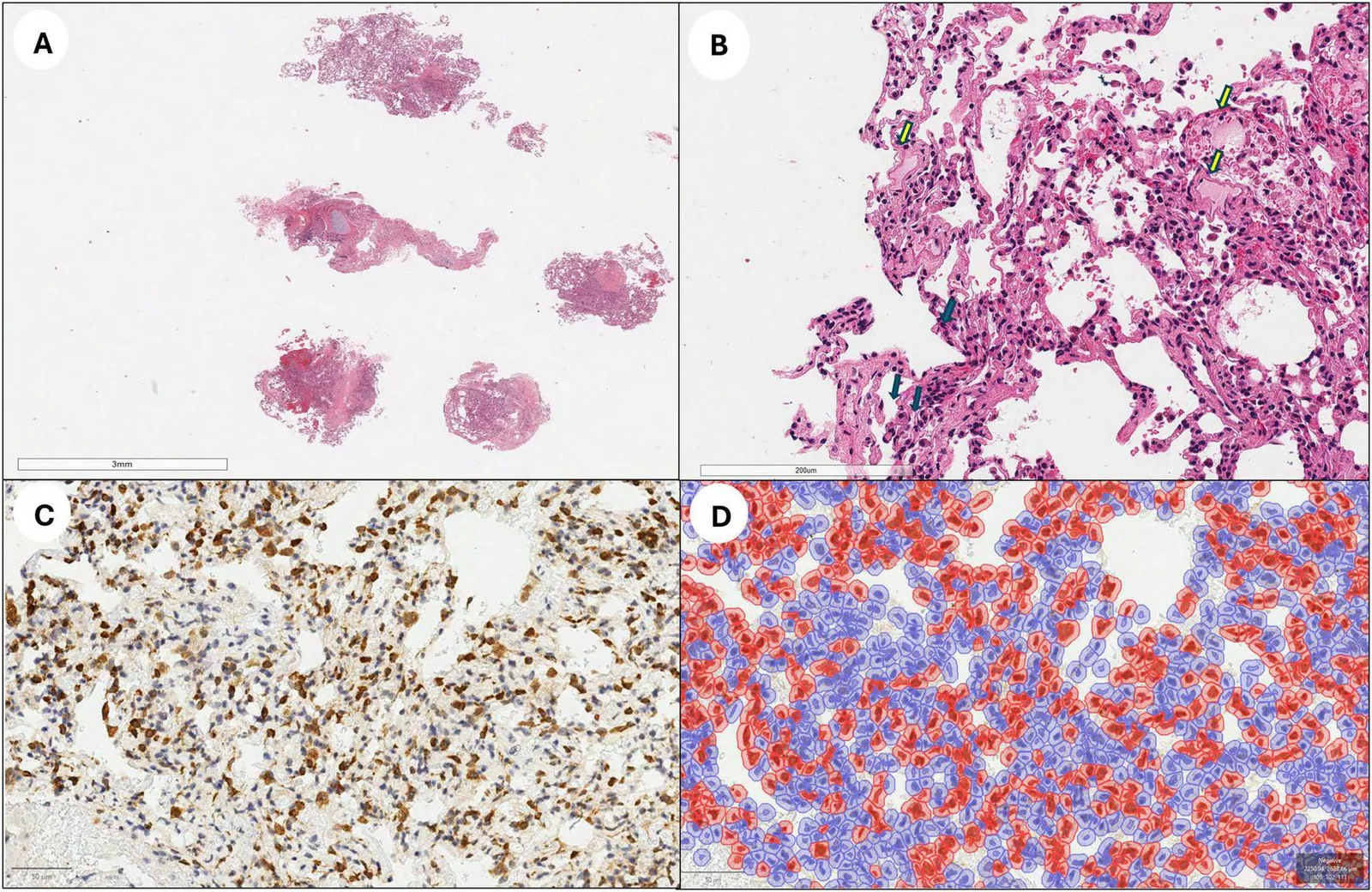
**(A)** Panoramic view showing adequate biopsy, with more than five samples including small and large airways (scale bar: 3 mm). **(B)** High magnification of one of the samples, showing marked interstitial edema with capillary inflammation and dilatation (yellow arrows for edema and capillary dilatation, blue arrows for capillary inflammation; scale bar: 200 μm). **(C)** Immunohistochemistry for p-S6RP. **(D)** QuPath elaboration of positive cells (in red) and negative cells (in blue) (scale bar: 50 μm). Cell detection: 13,000 cells with 9,100 negative and 3,900 positive (30.3% positivity rate, including macrophages, pneumocytes, and endothelial cells) (in absence of post-transplant complications, p-S6RP IHC shows only scattered positive macrophages, less than 5% of total cell count). IHC quantification was performed in QuPath (v. 0.6.0) using Optical density sum image (requested pixel size 0.2 μm), with nuclear segmentation (background radius 8 μm, median filter 1 μm, sigma 1.5 μm, nuclear area 10–400 μm^2^, threshold 0.1, max background intensity 2, split by shape). A 5 μm cell expansion was applied with measurements enabled, and outputs (positive/negative counts, %, cells/mm^2^) were computed within polygonal ROIs. TBBs, transbronchial biopsies; p-S6RP, phosphorylated S6 ribosomal protein; IHC, immunohistochemistry; ROIs, regions of interest.

Histological evaluation of the explanted lungs demonstrated widespread bronchiectasis, mucosal erosion, intense peribronchial and intraluminal inflammation with abscess formation, and interstitial hemorrhages, confirming end-stage cystic fibrosis. On 30 October 2023, on PTD 20, the first protocol TBB revealed moderate acute cellular rejection (ACR) (A3B0). In addition, moderate capillary inflammation (neutrophilic margination) and Bronchus-Associated Lymphoid Tissue (BALT) hyperplasia were observed, with negative C4d staining. p-S6RP expression was very high in all cell components. In the presence of circulating DSAs, these findings supported a diagnosis of mixed ACR and AMR, in agreement with the 2016 consensus report criteria (2). Following steroid treatment, repeat biopsy, performed on PTD 70, confirmed complete resolution of ACR. However, capillary inflammation and strong p-S6RP expression in endothelial cells, epithelial cells, and macrophages persisted, in absence of C4d staining, fulfilling the 2016 ISHLT criteria for subclinical probable AMR (2). In January (PTD 112), June 2024 (PTD 245), October 2024 (PTD 371), April 2025 (PTD 539), routine surveillance TBBs were performed and histological evaluation revealed no evidence of ACR but, despite the absence of clinical evidence of allograft dysfunction and of DSA-HLA, lung parenchyma was markedly affected by interstitial fibrosis, lymphomonocytic/granulocytic infiltrates, capillary inflammation, negative C4d and high p-S6RP values. These morphological features, in addition to a high p-S6RP expression in all the cell types (total score ≥ 7 in all TBB) were considered compatible with persistent antibody-mediated injury and raised suspicion for ongoing subclinical AMR (2). The discordance between these findings and the clinical–functional data was extensively discussed within the multidisciplinary team and prompted further in-depth investigation of the patient’s immunological profile. In July 2025 (PTD 637), when the patient was admitted to the ICU for respiratory failure, histological examination revealed severe DAD with hyaline membranes and high scores of p-S6RP in all three cell types. All these information are reported in Figures 1–3 and Table 1.

**TABLE 1:** Longitudinal histopathological, immunological, and clinical findings during post-lung transplantation follow-up.

Follow-up time (PTD)	Allograft dysfunction	Lung histology	Histological details	C4d immunostaining	HLA DSA	Non-HLA	AMR category
20	No	+	A3B0, capillary inflammation, BALT hyperplasia	−	+	+	Probable AMR + ACR
70	No	+	A0B0, capillary inflammation	−	+	+	Probable AMR
112	No	+	A0B0, capillary inflammation, fibrosis, interstitial lymphomonocytic infiltrates	−	−	+	Possible AMR
245	No	+	A0Bx, capillary inflammation, interstitial lymphomonocytic infiltrates	−	−	+	Possible AMR
371	No	+	A0B0, lymphocytic bronchopneumonia, capillary inflammation	−	−	+	Possible AMR
539	No	-	A0B0, interstitial lymphomonocytic infiltrates, microgranulomas, chronic bronchitis	−	−	+	Non AMR
637	Yes	+	A0B0, severe DAD	−	+	+	Probable AMR

### Immunological findings and follow up

**FIGURE 4:**
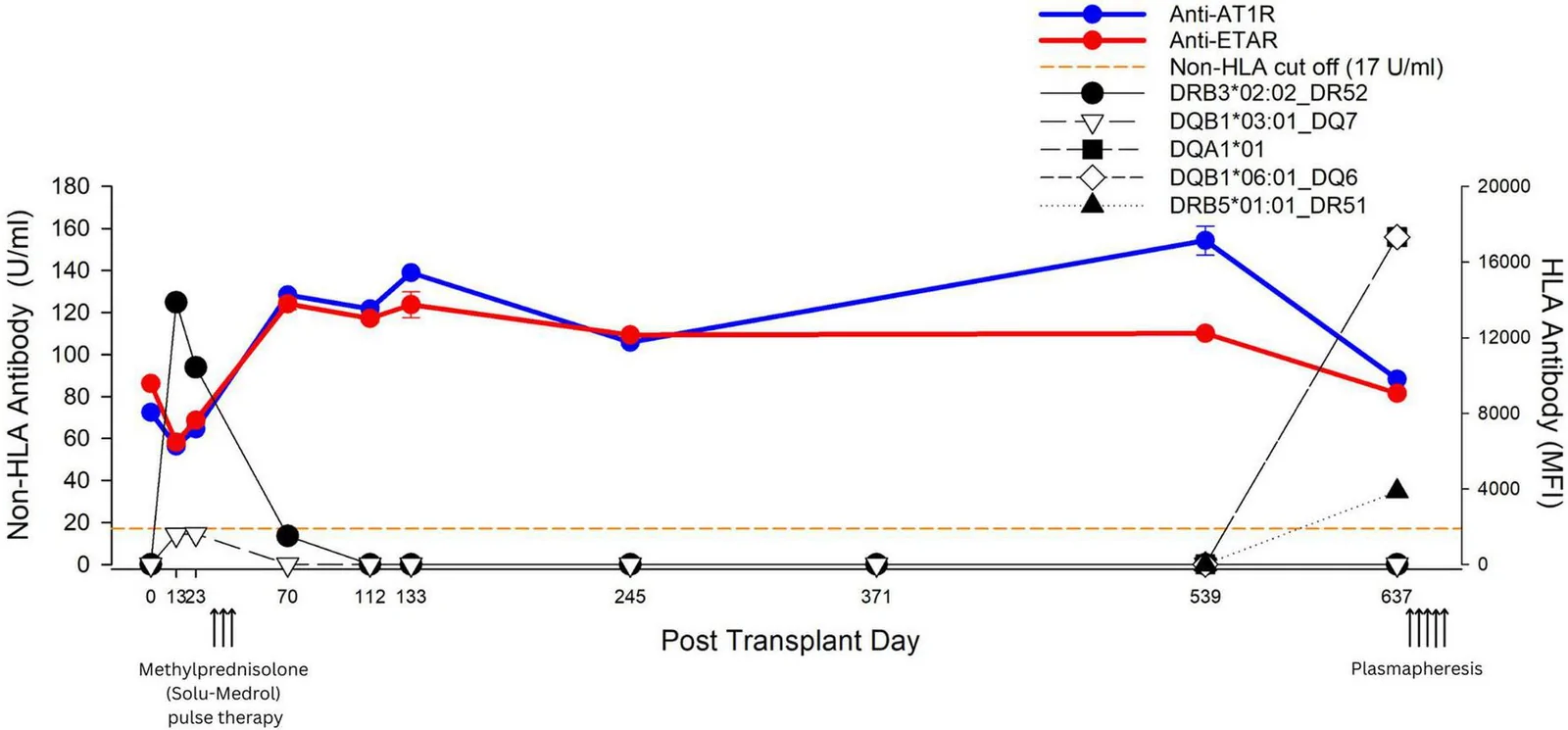
Longitudinal monitoring of anti-ETAR and anti-AT1R antibody levels in the pediatric lung transplant recipient. Anti-AT1R (blue) and anti-ETAR (red) antibody levels were measured over time in the serum samples available both pre-transplantation and during the post-transplant follow-up. The horizontal dashed line indicates the positivity cutoff for non-HLA antibodies. The fluctuations in DSA HLA antibodies are also reported (black lines). ETAR, endothelin type A receptor; AT1R, angiotensin II type 1 receptor; HLA, human leukocyte antigen; DSA, donor-specific antibodies.

No previous sensitizing events were reported in the lung recipient and no HLA antibodies were detected at the time of transplantation. An 8/8 HLA mismatch was observed between donor and recipient. Prior to transplantation, both T and B lymphocytes CDC cross matches were negative. The immunological follow-up is summarized in Figures 1, 4. The kinetics of both HLA-DSA and non-HLA antibodies are presented in Figure 4. Two weeks after transplantation, two *de novo* anti-HLA Class II DSA [anti-DRB3*02:02 with a mean fluorescence intensity (MFI) of 13871 and anti-DQB1*03:01, MFI: 1577] and three different HLA-Class I and Class II non-DSA were detected (anti-B*49:01, MFI: 422; anti-DQB1*03:02; MFI: 2942; anti-DQB1*03:03, MFI: 2352). On October 30, 2023 (PTD 20), at the time of the first TBB, repeat antibody monitoring showed persistently elevated DSA (anti-DRB3*02:02, MFI: 10434; anti-DQB1*03:01, MFI: 1613) and NDSA levels. Subsequent immunological monitoring on PTD 70, demonstrated a marked reduction in anti-DRB3*02:02 antibody levels (MFI: 1534), and repeat biopsy confirmed complete resolution of ACR. From January 2024 onwards (PTD 112), DSA and non DSA measurements remained negative. The case was discussed during weekly multidisciplinary team meetings, and a thorough investigation of non-HLA antibodies was strongly recommended. Given the persistence of histological signs raising suspicion for ongoing subclinical antibody-mediated injury despite negative HLA-DSA, an extended immunological assessment was performed. All pre- and post-transplant sera were tested for the non-HLA antibodies anti-AT1R and anti-ETAR antibodies. Surprisingly, pre-transplant serum was strongly positive for both anti-AT1R and anti-ETAR antibodies (72.5 ± 0 and 86.2 ± 0.4 U/mL, respectively). From PTD 23 onwards and immediately following the observed peak of HLA-DSA, a progressive increase in anti-AT1R and anti-ETAR antibody titers was observed. These non-HLA antibodies remained markedly elevated, showing only a slight decline by PTD 245 (June 2024) and persisting above pre-transplant levels. In July 2025, concomitantly with the appearance of severe graft dysfunction, a marked re-emergence of HLA DSA was detected (anti- DQB1*03:01, MFI: 20159). In addition, *de novo* appearance of additional HLA specificities including anti-DQB1*06 (MFI: 17323), anti-DQA1*01 (MFI: 17323), and anti-DRB5*01:01 (MFI: 3871) was observed. The measurement of non-HLA anti-AT1R and anti-ETAR antibodies, demonstrated persistently elevated levels, remaining comparable to those measured in the pre-transplant period (Figure 4).

## Discussion

This case highlights the diagnostic complexity of lung AMR, particularly when clinical, immunological, and histopathological findings do not fully align. The evolution of pathological features over time, together with the disappearance of HLA-DSA, emphasizes the limitations of conventional diagnostic approaches and the need for a multidisciplinary strategy integrating expanded immunological assessment. In this context, p-S6RP has emerged as a promising complementary biomarker of antibody-mediated endothelial injury, showing encouraging diagnostic performance in LTx (10–13) and other solid organ transplants (14, 15). In our patient, diffuse p-S6RP overexpression provided an important clue of ongoing graft injury despite undetectable HLA-DSA, supporting further investigation that ultimately revealed persistent non-HLA antibody positivity. Previous studies by Lunardi et al. (12) demonstrated that p-S6RP expression may improve AMR discrimination compared with conventional markers such as C4d, although it should not be interpreted as an isolated diagnostic criterion. Indeed, at our center, p-S6RP is interpreted within an integrated clinical, microbiological, pathological, and immunological framework. This case suggests that incorporating p-S6RP into the diagnostic work-up may help identify complex AMR phenotypes and prompt recognition of non-HLA antibody-mediated mechanisms that may otherwise remain undetected.

Thus, persistent p-S6RP expression despite HLA-DSA clearance supports the hypothesis that mTOR pathway activation contributes to AMR independently of complement activation. Although HLA-DSA are the more frequent mediators of AMR, increasing data link non-HLA antibodies to graft injury in kidney and heart transplantation and, more recently, in LTx, where they have been associated with CLAD and graft decrease of functionality (6, 16).

Antibodies targeting endothelial G-protein–coupled receptors (GPCRs), such as AT1R and ETAR, have been associated with lower freedom of AMR, cellular rejection, *de novo* DSA formation, and reduced graft survival (5, 17). In our case, both anti-AT1R and anti-ETAR antibodies were elevated before transplantation, persisted throughout follow-up, and paralleled the evolution of histological injury, suggesting that their persistence after transplantation and temporal association with sustained histological evidence of endothelial injury raise the possibility that they acted as contributors or amplifiers of graft injury. Nevertheless, this interpretation remains speculative. While our findings support an association between persistent non-HLA antibodies and ongoing AMR-like pathology, they cannot establish a direct causal relationship.

Notably, antibody levels exceeded the assay’s upper limit of detection, requiring serum dilution for accurate quantification. The exceptionally high titers likely reflect the combined influence of the patient’s young age and the underlying disease. Indeed, pediatric recipients have been reported to exhibit higher circulating anti-AT1R antibody levels than adults, suggesting age-related differences in immune responsiveness (18). Patients with cystic fibrosis or idiopathic pulmonary fibrosis have been reported to exhibit higher levels of anti-AT1R and anti-ETAR antibodies than patients with other lung diseases, possibly because of chronic airway inflammation and recurrent infections (5, 19). Although these factors may contribute to endothelial activation and p-S6RP upregulation, they are unlikely to fully explain the persistent endothelial injury observed in our patient. Once initiated, endothelial activation may become self-sustaining in the presence of autoantibodies, independently of the original trigger. Consistently, both p-S6RP overexpression and circulating anti-AT1R/anti-ETAR antibodies persisted throughout follow-up despite resolution of the initial infectious and ischemic insults, supporting an ongoing antibody-mediated mechanism. Nevertheless, although these antibodies may reflect enhanced endothelial immune activation, their quantitative relationship with pathogenicity and clinical outcome remains poorly defined in LTx, and no validated threshold predictive of AMR or graft failure is currently available. The antibody kinetics observed in our patient are consistent with previous evidence suggesting that non-HLA antibodies may contribute to graft injury and precede the development of HLA-DSA, with potential prognostic implications (20). Although speculative, anti-AT1R and anti-ETAR antibodies may promote endothelial activation, microvascular inflammation, and tissue remodeling through receptor-mediated signaling pathways (21, 22). Their ability to activate endothelial mTOR signaling may explain the persistent p-S6RP expression and capillary inflammation observed despite HLA-DSA clearance, in line with previous reports linking non-HLA stimulation of mTOR pathways to impaired repair mechanisms and transplant vasculopathy (23). While GPCR antibody-associated rejection phenotypes have been described mainly in kidney transplantation (24), their relevance in LTx remains poorly characterized. Our findings suggest that this mechanism may represent an underrecognized form of antibody-mediated endothelial injury in lung allografts.

Despite growing interest, non-HLA antibody testing remains limited by the lack of standardized, validated assays and the absence of consensus thresholds defining clinically relevant positivity. Therefore, routine pre-transplant screening is not currently recommended. However, targeted assessment may be considered in selected high-risk patients, including pediatric recipients, patients with cystic fibrosis or idiopathic pulmonary hypertension, autoimmune backgrounds, retransplantation, or unexplained endothelial activation. The identification of non-HLA antibodies before transplantation also raises important therapeutic questions. In our case, conventional anti-rejection therapies appeared insufficient to achieve sustained control of antibody levels. Although evidence remains limited, strategies including antibody removal, anti-plasma cell therapies, and receptor-targeted approaches have been proposed as potential options (8, 25, 26). Further studies are required to define their role in LTx. In this context, persistent p-S6RP activation may also support the potential relevance of targeting mTOR signaling, as previously suggested in relation to anti-AT1R and anti-ETAR-mediated pathway activation (23). However, everolimus was not introduced as a targeted treatment for persistent p-S6RP activation or subclinical AMR, since evidence supporting this strategy is currently lacking (27). The switch from mycophenolate mofetil to everolimus was performed only at PTD 539, as an optimization of maintenance immunosuppression in response to persistent interstitial lymphocytic inflammation in the absence of infection, rather than as a treatment specifically targeting the p-S6RP/mTOR pathway. Indeed, at present, p-S6RP remains a promising but still exploratory biomarker, and its role in guiding targeted immunosuppressive strategies has yet to be established. Nevertheless, mTOR inhibition could represent a potential therapeutic strategy in the future, particularly if objective and reproducible methods for quantifying pathway activation become available (27). The development of standardized quantification approaches—potentially supported by artificial intelligence–based algorithms—may help refine patient selection and enable more personalized treatment strategies targeting non-HLA–mediated mechanisms.

A major diagnostic challenge in this case was distinguishing AMR-related capillary inflammation from infection-associated injury. Despite extensive microbiological investigations, including repeated BAL analyses, cultures, and specific stains, no active infection was identified to explain the persistent microvascular inflammation. Although chronic MDR Pseudomonas colonization, CMV infection, bronchial stenosis, and repeated bronchoscopic interventions may have contributed to endothelial activation, the persistence of these abnormalities despite HLA-DSA clearance and ACR resolution, together with sustained anti-AT1R and anti-ETAR positivity, supported the multidisciplinary diagnosis of ongoing antibody-mediated endothelial injury.

Finally, the patient’s and family’s decision to decline re-listing for transplantation was a major determinant of the final outcome and underscores the complex clinical, ethical, and psychosocial challenges influencing retransplantation decisions in pediatric LTx recipients.

This case illustrates the multifactorial nature of AMR in LTx and provides valuable longitudinal insights into a still underreported pediatric population. Its main strength lies in the integration of clinical, pathological, and immunological data, allowing a dynamic assessment of graft injury over time. In particular, this case reinforces that AMR diagnosis cannot rely on a single parameter but requires the interpretation of complementary findings within a multidisciplinary framework (28). The persistence of p-S6RP expression despite HLA-DSA clearance provided both diagnostic and mechanistic insight into ongoing endothelial injury, while extended antibody profiling revealed a potential contribution of non-HLA antibodies, particularly anti-AT1R and anti-ETAR, to the evolution of graft dysfunction. These findings should be interpreted considering the limitations of a single-case report, including the absence of standardized non-HLA antibody assays and targeted therapeutic strategies. Nevertheless, this case highlights three critical challenges in pediatric LTx: recognizing AMR phenotypes in the absence of detectable HLA-DSA, identifying complementary biomarkers of antibody-mediated endothelial injury, and understanding the emerging role of non-HLA antibodies in graft dysfunction. Integrating histological, immunological, and molecular information may improve the characterization of complex AMR phenotypes that remain difficult to classify using current diagnostic criteria alone.

## Data Availability

The original contributions presented in this study are included in this article/supplementary material, further inquiries can be directed to the corresponding author.
